# Editorial: Experimental and innovative approaches to multi-target treatment of Parkinson's and Alzheimer's diseases - Volume II

**DOI:** 10.3389/fnins.2023.1171866

**Published:** 2023-03-24

**Authors:** Maria A. Tikhonova, Hung-Ming Chang, Sandeep Kumar Singh

**Affiliations:** ^1^Laboratory of the Experimental Models of Neurodegenerative Processes, Scientific Research Institute of Neurosciences and Medicine (SRINM), Novosibirsk, Russia; ^2^Department of Anatomy and Cell Biology, School of Medicine, College of Medicine, Taipei Medical University, Taipei, Taiwan; ^3^Indian Scientific Education and Technology Foundation, Lucknow, India

**Keywords:** neurodegeneration, experimental therapy, neuromodulation techniques, animal models, multi-interventions, neuroinflammation, network pharmacology, machine learning

Pathogenesis of neurodegenerative disorders including Alzheimer's disease (AD) and Parkinson's disease (PD) has complicated nature. Many factors and processes contribute to the disease onset and progression. In addition to neurotoxicity of protein aggregates, neurodegenerative disorders involve various pathological processes, e.g., oxidative stress, neuroinflammatory response, disturbed neurotrophic function and neurogenesis, synaptic and neurotransmission dysfunction, ion disbalance, that tightly interact and overlap. Thus, multipurpose or multi-target therapy aimed at various important pathogenetic hubs is a modern trend regarded as a promising strategy for AD and PD treatment. The aim of this Research Topic was to provide an updated overview on multi-targeted therapy for AD and PD and related issues. In the Volume II, we continue collecting the evidence of multifactorial nature of neurodegenerative disorders and multi-target approach in their current experimental correction.

According to PubMed search, the last year brought 200+ publications related to multi-target or multipurpose treatment of neurodegenerative pathology. Many novel multipotent drugs were introduced, i.e., 4-oxo-N-4-diphenyl butanamides (reversible selective monoamine oxidase B inhibitors with anti-acetylcholinesterase activity) for AD treatment (Jana et al., 2023); benzothiazole derivatives (histamine H3 receptor ligands targeted at acetylcholinesterase, butyrylcholinesterase, and monoamine oxidase B enzymes) for AD therapy (Hafez et al., 2023); vanilloid-triazole conjugates as dual inhibitors of acetylcholinesterase and Aβ aggregation for AD treatment (Elsbaey et al., 2023); N-benzyl piperidine derivatives as potent histone deacetylase and acetylcholinesterase inhibitors for AD therapy (Qin et al., 2023); 5,6-dimethoxy-indanone-chalcone-carbamate hybrids as dual inhibitors of acetylcholinesterase and inflammation against AD (Liu et al., 2022); benzimidazole arylhydrazones as antioxidant radical-scavenging agents and MAO-B inhibitors with neuroprotective properties for PD therapy (Anastassova et al., 2022); chalcones as enzyme inhibitors (monoamine oxidase B, catechol-O-methyltransferase, and acetylcholinesterase), inhibitors of α-synuclein aggregation, showing anti-neuroinflammatory activity (inhibition of iNOS or activation of Nrf2 signaling), as well as antagonists of adenosine A1 and/or A2A receptors for PD treatment (Krolicka et al., 2022); resveratrol analogs as dual inhibitors of monoamine oxidase B and carbonic anhydrase VII against neurodegenerative disorders (Carradori et al., 2022).

Moreover, multi-target drugs and combinations are actively studied in clinical trials. For example, disaccharide trehalose is a multipotent agent aimed at autophagy induction and lysosome and autophagosome biogenesis pathways as well as possessing chaperone-like effect on proteins, anti-inflammatory effect and antioxidant activity (Pupyshev et al., 2022). In 2021, Bioblast Pharma Ltd. (Israel) and its present owner Seelos Therapeutics (USA) announced a large project on the trehalose treatment of neurodegenerative diseases accompanied by accumulation and aggregation of proteins (Treatment of protein aggregation in myopathic and neurodegenerative diseases by parenteral administration of trehalose, https://patents.google.com/patent/EP2994145A2/en; accessed 18 February 2023). In particular, clinical trials of trehalose have been announced regarding the treatment of AD (Mashhad University of Medical Sciences, Iran, since August 2020, phase 1) (https://adisinsight.springer.com/drugs/800039496; accessed 18 February 2023). Here, Reich and Hölscher masterly presented mimetics of glucagon-like peptide 1 (GLP-1) as promising multi-target agents. They provide an accurate in-depth review of the existing evidence about the neuroprotective pathways that are induced following the activation of GLP-1 receptors in neurons, microglia, and astrocytes. Noteworthy, several clinical trials of GLP-1 mimetics in patients with AD and PD have been conducted and have shown good effects. Challenges and prospects of clinical trials of GLP-1 receptor agonists in neurodegenerative disorders are also discussed.

Another trend is the spread of cutting-edge technologies of network pharmacology, pharmacoinformatics tools, molecular docking, and machine learning in the field. For example, a method of network pharmacology including a molecular docking technology revealed multi-targets and multichannels of anti-AD activity for two Chinese medicines (*Fibraurea recisa Pierre* and Curcumin, a major ingredient of traditional Chinese medicine, Curcuma Longa) (Wang et al., 2022; Wu et al., 2022). Bai Chan Ting, a traditional Chinese medicine prescription, consisting of three herbs (*Acanthopanax senticosus, Paeonia lactiflora*, and *Uncaria*), was found to produce multi-target and multi-pathway effects against PD-like pathology in transgenic mice using an integrated strategy of network pharmacology and brain metabolomics (Zhang et al., 2022). In the present article collection, Xu et al. clarified the pharmacodynamic mechanisms of Qin-Zhi-Zhu-Dan Formula in treating AD-like pathology and revealed its neuroprotective effects through the regulation of TNFR1-ERK1/2-NF-κBp65 signaling pathway combining animal experiments with network pharmacology analysis [namely, Gene Ontology (GO) enrichment analysis and Kyoto Encyclopedia of Genes and Genomes (KEGG)].

Resting tremor is a characteristic symptom of idiopathic PD. Although its classification is an important clinical issue, its current evaluation in clinical practice is rather subjective and limited while many studies propose novel approaches. Channa et al. suggested classification of resting tremor severity using machine learning with resampling techniques and discussed related studies in the field.

In parallel to the mentioned approaches, non-pharmacological techniques targeted at the modulation of the brain activity such as deep brain stimulation (DBS) are applied to control drug-resistant symptoms of PD and AD. Noteworthy, current practice may involve multiple targets for neuromodulation in the brain. In a case report by Li et al., combined therapy of bilateral sub-thalamic nucleus DBS and spinal cord stimulation was introduced. The method significantly improved motor function in a patient with multiple system atrophy with predominant parkinsonism. A review by Brak et al. discusses methods of transcranial electric current stimulation for the treatment of cognitive impairment in PD patients. Transcranial alternating current stimulation is a relatively new neuromodulation technology aimed at changing the functional oscillatory activity of the brain cortex and perhaps modulating neuroplasticity processes.

And last but not least, Gao et al. reported about a case of early-onset PD caused by a novel compound heterozygous mutation in *PLA2G6* gene encoding phospholipase A2 type VI.

Taken together, the papers collected in this Issue present the most recent knowledge and experimental evidence about the multi-target approach for therapy of neurodegenerative disorders and offer a new perspective and interesting hypotheses on this topic.

## Author contributions

All authors listed have made a substantial, direct, and intellectual contribution to the work and approved it for publication.
